# A comparative study of bronchopulmonary slowly adapting receptors between rabbits and rats

**DOI:** 10.14814/phy2.15069

**Published:** 2022-03-28

**Authors:** Ping Liu, Igor N. Zelko, Jerry Yu

**Affiliations:** ^1^ Department of Medicine University of Louisville Louisville Kentucky 40292 USA; ^2^ Robley Rex VA Medical Center Louisville Kentucky 40206 USA

**Keywords:** control of breathing, pulmonary stretch receptor, vagal afferents

## Abstract

Pulmonary mechanosensory receptors provide important inputs to the respiratory center for control of breathing. However, what is known about their structure–function relationship is still limited. In these studies, we explored this relationship comparing bronchopulmonary slowly adapting receptor (SAR) units in rabbits and rats. In morphological studies, sensory units in tracheobronchial smooth muscle labeled with anti‐Na^+^/K^+^‐ATPase (α3 subunit) were found to be larger in the rabbit. Since larger structures may result from increased receptor size or more numerous receptors, further examination showed receptor size was the same in both species, but more receptors in a structure in rabbits than rats, accounting for their larger structure. In functional studies, SAR units were recorded electrically in anesthetized, open‐chest, and artificially ventilated animals and responses to lung inflation were compared at three different constant airway pressures (10, 20, and 30 cmH_2_O). At each level of the inflation, SAR discharge frequencies were found to be higher in rabbits than rats. We conclude that a relatively larger number of receptors in a sensory unit may be responsible for higher SAR activities in rabbit SAR units.

## Graphical abstract

Comparison of pulmonary mechanosensory structures and electrical activities between rabbits and rats. Mechanosensory units have more sensors and activity in rabbits than in rats. Electrical activity of the sensory unit is directly related to the number of sensors.

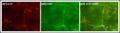

## 1 INTRODUCTION

**FIGURE 1:**
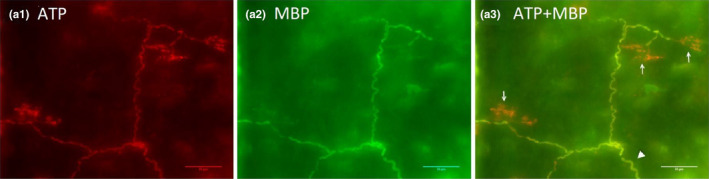
A double staining approach to illustrate SAR sensory receptors and structures identified in rabbit airways. The axon demyelinated before reaching the end‐formation. The receptor is identified as the end‐formation extended beyond the myelin (pure red portions indicated by white arrow in A3). A sensory structure usually contains several receptors connected by a parent axon (indicated by an arrow head)

Airway sensory receptors provide constant information to the respiratory centers that is vital in control of breathing. Afferent inputs arising from these sensors regulate not only respiratory pattern, but also affect outcomes in cardiopulmonary diseases, such as heart failure, acute respiratory distress syndrome (ARDS), chronic obstructive pulmonary disease (COPD), and asthma (Lee & Yu, 2015). Until now, at least six different types of bronchial pulmonary airway sensory receptors have been identified (Yu, 2020). Among them, slowly adapting receptors (SARs) are most extensively studied morphologically and physiologically; they are the focus of this study. Three sensory terms are employed herein: receptor, structure, and unit (Liu et al., 2016). A receptor is an encoder, the basic device that generates action potentials. Morphologically, a receptor is comprised of expanded end‐formations after axon demyelination. Action potentials are generated at the first node of the myelin sheath. A sensory structure is a portion of a sensory unit observed under a microscope that usually contains several receptors connected by a parent axon. A sensory unit is a functional unit that transmits action potentials to the central nervous system. Morphologically, a sensory unit may house more than one sensory structure. Figure 1 demonstrates sensory receptors and structures identified by a double staining technique, illustrating these definitions. In the figure Na^+^/K^+^‐ATPase (α3) stains a structure in the sensory unit (red, image A1), and myelin basic protein (MBP) stains the myelin sheath (green, image A2) and shows yellow (co‐staining, image A3) in the composite.

Mechanosensitive SARs located in airway smooth muscle in the membranous posterior wall of the trachea (Sant'Ambrogio, 1982; Widdicombe, 2001) respond more to transverse than longitudinal stretching of the wall (Bartlett et al., 1976; Widdicombe, 1954). Morphologically, sensory receptor structures are found in tracheal smooth muscle under light microscopy (Baluk & Gabella, 1991; Larsell, 1922; Yamamoto et al., 1994) or electron microscopy (Krauhs, 1984; Yamamoto et al., 1994). Although the morphology and function of airway sensory receptors have been intensively investigated for almost a century, what is known of their structure‐function relationships remains limited. With advances in technology, airway sensory structures have been examined in detail and can be evaluated more objectively (Yu, 2005). Using antibodies against Na^+^/K^+^‐ATPase (α3 subunit), SAR structure has been identified in both rabbits (Yu et al., 2003) and rats (Yu et al., 2004). The structure and size of SARs can be compared, for example, between large and small airways (Liu et al., 2016). While the receptor structure is larger in the large airway than the small airway, receptor sizes are similar. Therefore, the receptor structure in the large airway contains more receptors. This provides an opportunity to delineate the receptor structure–function relationship, which is important in understanding the roles of sensory receptors in pulmonary physiology and pathophysiology. Although structures of SARs in the airway have been examined in rabbits, rats, and other species (Yu, 2005), there is no comparison made between them. Since electrical recording and histochemical labeling techniques are available for rabbits and rats in our laboratory, both species have a strong Hering–Breuer inflation reflex, and they represent medium‐ and small‐sized animals, we carried out a series of comparative studies between rabbits and rats. To delineate SAR structure–function relationships, we tested whether the size and discharge frequencies of SARs are the same or different between the two species?

## 2 METHODS

### 2.1 Animal preparation

Male New Zealand white rabbits (1.5–2.0 kg) and Wistar rats (280–360 g) were used in current studies, in conformance with the Guide for the Care and Use of Laboratory Animals published by the United States National Institutes of Health (NIH Publication No. 85–53) and approved by the Institutional Animal Care and Use Committee at University of Louisville and the Robley Rex VA Medical Center.

### 2.2 Electrical recording of sensory unit activity

For functional studies, we used the single‐fiber recording technique (Liu & Yu, 2013). Briefly, rabbits (20% urethane at 1 g/kg, i.v.) and rats (sodium pentobarbital at 40–50 mg/kg, i.p.) were anesthetized. A midline incision was made to expose the trachea and vagus nerve. The trachea was cannulated for mechanical ventilation (Harvard ventilator, model no. 683). The chest was opened widely. Animals were ventilated at 8 ml/kg body weight. Positive‐end‐expiratory pressure (PEEP) was maintained by placing the expiratory outlet under 2–4 cmH_2_O. The vagus nerve (either right or left) was separated from the carotid sheath, placed on a dissecting platform, and covered with mineral oil. A small slip was isolated from the vagus nerve, and placed on recording electrodes with the main trunk of the vagus nerve left intact. The electrodes were connected to a high‐impedance probe (Grass HIP5) from which the output signal was amplified (Grass P511). Signals from the unit activity were displayed on an oscilloscope and recorded along with airway pressure by a Dash IV thermorecorder (Astro‐Med). SARs were identified by their discharge pattern and adaptation index (<20%). Impulse frequency was counted by a rate meter at a bin width of 0.1 s. Unit responses to lung inflation at different constant pressures (10, 20, and 30 cmH_2_O) were examined.

### 2.3 Immunochemical staining of sensory structures

In structural studies, double‐stain antibodies were applied (Na^+^/K^+^‐ATPase α3 subunit, Adriaensen et al., 2006; Mazzone et al., 2009; Matsumoto et al., 2006 and myelin basic protein, Brouns et al., 2004; Yokoyama et al., 2019), using previously reported techniques to examine sensory structures (Liu et al., 2016; Yu et al., 2004). Animals were euthanized by deep anesthesia (by repeating the initial anesthetic dose listed above), followed by an overdose of saturated KCl intravenously to arrest the heart. Airway tissues from the trachea and bronchi were obtained and fixed overnight in a 0.1 M phosphate‐buffered (PB) solution containing 4% paraformaldehyde (at pH 7.4). The preparation was then placed in a washing buffer (0.4% Triton X‐100 in 0.1 M PB) that was changed hourly for 6 h, followed by incubation in a blocking solution (containing 5% normal serum, 3% bovine serum albumin in washing buffer) for 2 h. Then, the preparation was incubated overnight at 4℃ with a monoclonal antibody (Anti‐Na^+^/K^+^‐ATPase, α3 subunit; Biomol, Cat# SA‐247; diluted to 1:200) (Table 1). Antiserum was washed from preparations using PB solution and tissue blocks were incubated overnight with cy3‐labeled donkey anti‐mouse immunoglobulin G (Jackson Immuno Research, diluted at 1:100–1:200). Some segments were also incubated with chicken polyclonal anti‐myelin basic protein (MBP) (AVES Labs, Inc., Cat# MBP; diluted to 1:100) (Table 1) and then treated with Alexa Fluor@488 goat anti‐chicken IgG (Invitrogen corporation; diluted to 1:500) for 60–120 min at room temperature. After washing with PB solution, the tissue was mounted on a glass slide with mount medium and examined microscopically (Olympus system; Model 1X71). Images were taken and digitally analyzed with the software (Image‐Pro Plus). As controls, attempts were made to stain tissues omitting primary antibody to rule out nonspecific staining, and omitting secondary antibody to rule out autofluorescence. Negative results were found in the antibody omission studies, confirming the quality of the staining method.

**TABLE 1:** Primary antibodies used

Antibody	Host	Source	Dilution	RRID
Anti‐MBP	Chicken	AVES Lab	1:100	RRID :A B_2313550
Anti‐ATPase α3	Mouse	MOLBIOL Intl	1:200	RRID :A B_2051956

### 2.4 Morphometric analysis

For quantification of sensory structure sizes in the airways, we measured two‐dimensional projection area to assess the receptor and structure sizes expressed in square micrometers using Image‐Pro Plus (MediaCybernetics) software, standardized by an internal scale bar in each acquired fluorescent image. Receptor area was measured manually by carefully outlining the shape of the structure. To verify the measurement reproducibility, morphometric quantification was performed by two independent investigators. Images with high‐quality fluorescent structures (clean background, with the receptor structures clearly labeled) were used for image analysis.

### 2.5 Statistical analysis

Group data are expressed as mean ± SE. Two‐group comparisons were made by Independent samples *t*‐test, and three‐group comparisons were conducted by repeated measurements one‐way analysis of variance using GB‐STAT. A value of *p* < 0.05 was considered to be statistically significant.

## 3 RESULTS

In the current studies, conventional histochemical staining and electrical recording techniques were employed to examine and compare airway sensory receptor structure and function in rabbits and rats.

### 3.1 Sensory structures

**FIGURE 2:**
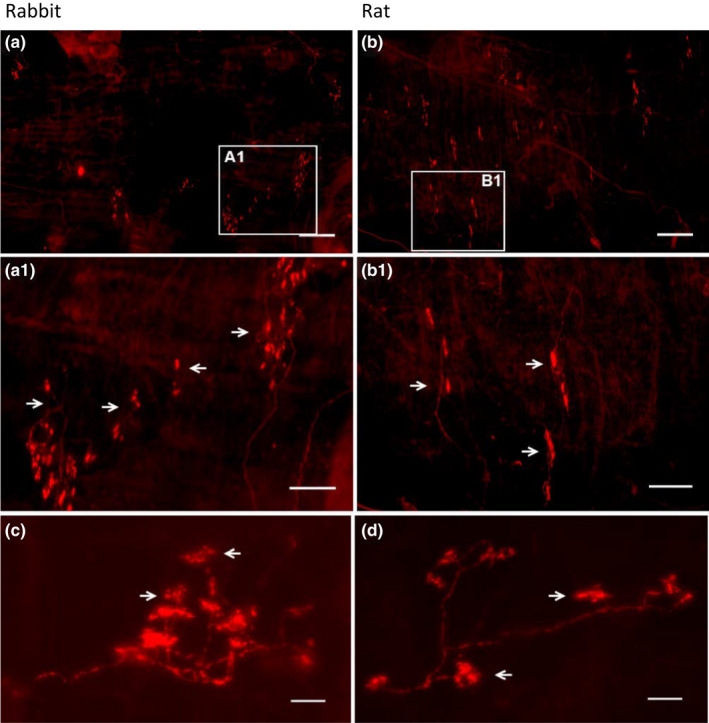
Comparison of sensory structures [stained with Na^+^/K^+^‐ATPase antibody] in the rabbit (A, A1, and C) and rat (B, B1, and D) airway. Images A1 and B1 are the amplified white squares in A and B. 4 and 3 sensory structures are indicated by white arrows in A1 and B1, respectively. C and D show the details of a sensory structure. The parent axon gives off some branches with many knob‐like extensions. Each knob indicated by a white arrow is a receptor. Scale bars are 500, 200, and 50 µm for top, middle, and bottom images, respectively

**FIGURE 3:**
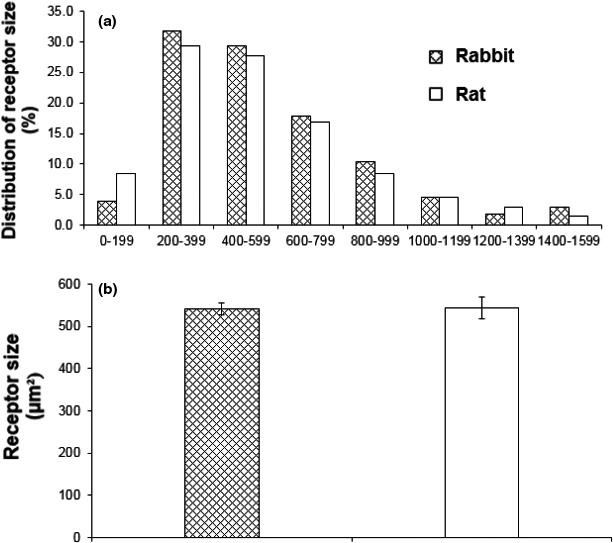
Comparison of receptor size in airways of the rabbit and rat. The distribution of receptor sizes was similar in both groups, and the size peaks at 200–600 µm² (a), with the same averaged receptor sizes (b)

**FIGURE 4:**
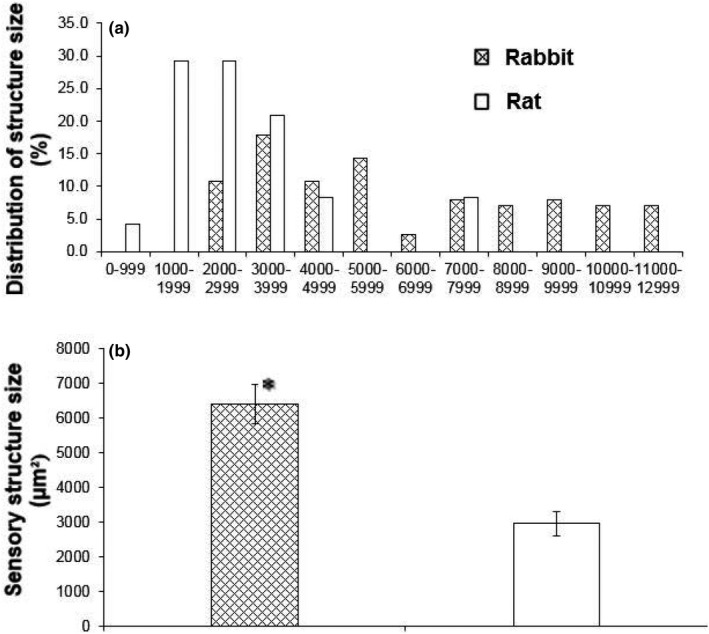
Comparison of sensory structure sizes in rabbit and rat airways. The distribution pattern demonstrates that rat sensory structures are concentrated at small size end (1000–3000 µm²), while rabbit sensory structures are more scattered at the large end (a), resulting in larger averaged structure size (b)

**FIGURE 5:**
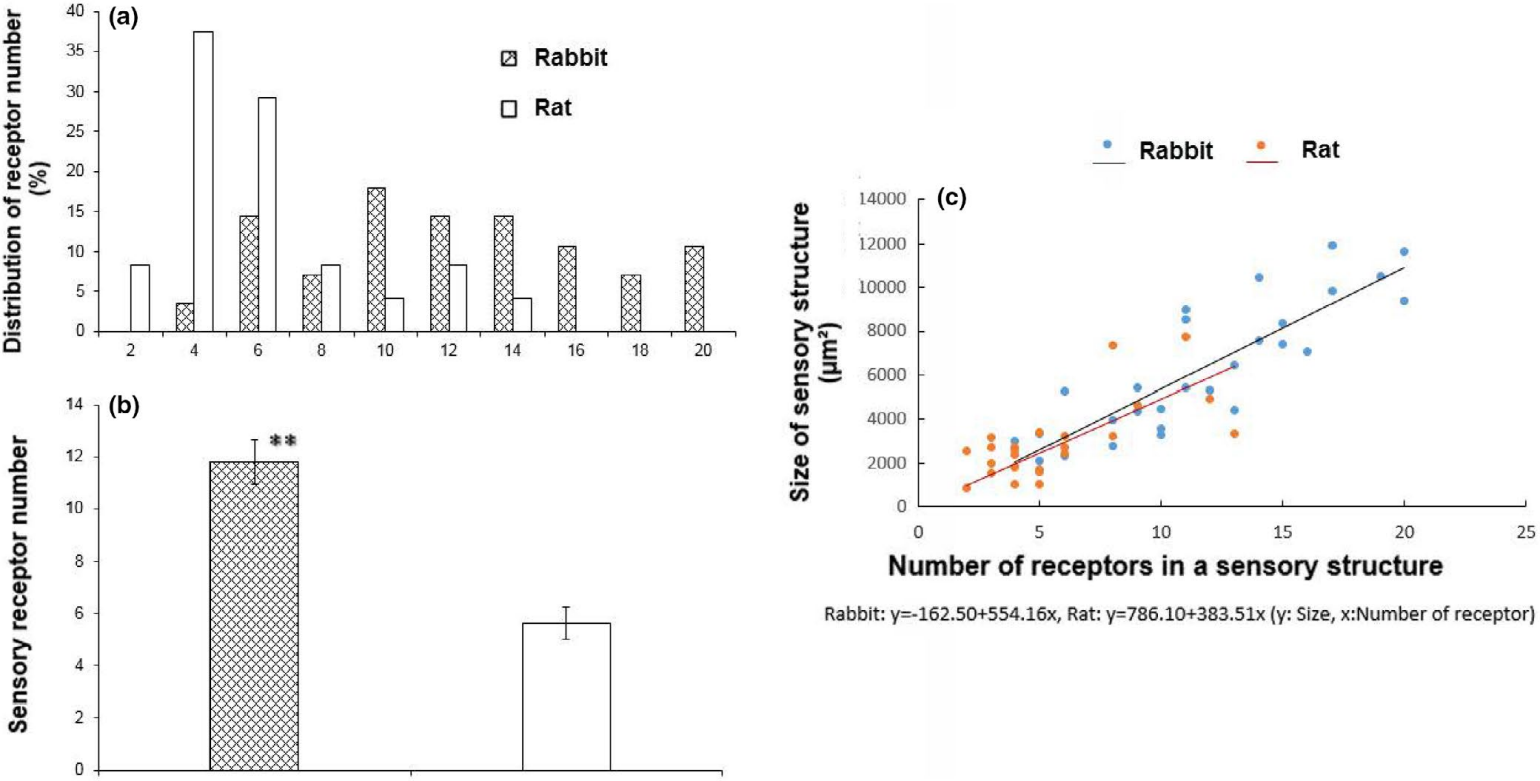
Relationship between structure size and the number of receptors. Although they overlap (a), sensory structures have more receptors in the rabbit than rat (b). Slopes were the same in rabbits and rats (c)

Overall, the morphology of the sensory structures showed a parent axon giving off branches to form knob‐like or leaf‐like extensions (Figure 2). The mean size of the airway receptors (projection area) was the same in rabbits (541.1 ± 14.4 µm²; *n* = 330) and rats (543.4 ± 26.7 µm²; *n* = 130) (*p* = 0.9405) (Figure 3). Both distributions skewed leftward, with median values of 487.7 and 458.6 µm² in rabbits and rats, respectively. Sensory structure size, however, was larger in rabbits (6377.5 ± 562.6 µm², *n* = 28) than rats (2943.4 ± 356.8 µm², *n* = 24) (*p *< 0.0001) (Figure 4). Correspondingly, the number of receptors in a structure is more numerous, c.f., 11.8 ± 0.9/structure, *n* = 28, and 5.6 ± 0.6/structure, *n* = 24 (*p *< 0.0001) (Figure 5). That is, sensory structures are more complex in rabbits owing to more receptors in each structure. The range of receptor numbers in sensory structures is from 4 to 20 in rabbits and from 2 to 13 in rats. The relationship is best illustrated by plotting structure size against the number of receptors (Figure 5c).

### 3.2 Sensory electrical activities

**FIGURE 6:**
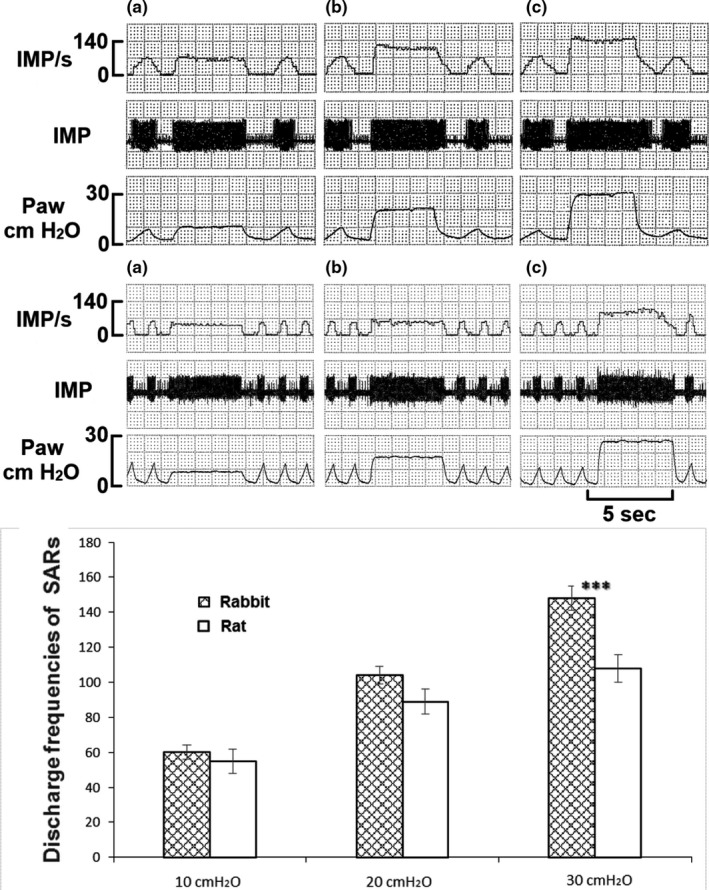
SAR unit responses to different levels of lung inflation at stepwise increments (a, B, and C are 10, 20, and 30 cmH_2_O, respectively) in rabbits (up) and rats (low). The traces are IMP/s, impulses (action potentials) per second; IMP, original recording; Paw, airway (cmH_2_O). Bottom part is grouped data. The discharge frequencies were higher in rabbits than in rats. ***indicates *p *< 0.05

SAR activities were assessed by single‐unit recording in the rabbit and rat. SAR responses to constant pressure inflation of the lungs at different levels were recorded and compared. Under resting conditions, SARs had similar basal discharge frequencies, with averaged mean and peak of 25 ± 3 and 79 ± 6 imp/s in rabbits and 30 ± 3 and 70 ± 5 imp/s in rats (*p *> 0.05). Discharge frequency increased with increasing inflation pressure in both (Figure 6). The peak frequencies were 60 ± 4, 104 ± 45, and 148 ± 7 imp/s for rabbits (*n* = 34) and 55 ± 7, 89 ± 7, and 108 ± 8 imp/s for rats (*n* = 31) at pressures of 10, 20, and 30 cmH_2_O, respectively. However, incremental discharge frequencies were higher in rabbits, reaching statistical significance at 30 cmH_2_O inflation, *p *< 0.05 (Figure 6). Thus, we calculated linear regressions of impulse frequencies (imp/s) against airway pressures (cmH_2_O) (Rabbit *y* = 11.4 + 3.88*x*; Rat *y* = 24.2 + 2.21*x*). The slope was steeper in the rabbit than in the rat. The difference was statistically significant (*p *< 0.01).

## 4 DISCUSSION

It is of interest to know how morphological differences in sensory structures may account for behavioral differences. Identifying such relationships would potentially lead to better understanding the underlying mechanisms of sensory behavior. However, no comparative studies focus on this issue. To our knowledge, this study is the first attempt to explore pulmonary sensory unit activation mechanisms by comparing the structure and function of SARs in the rabbit and rat lungs. Our results demonstrate that while sensory receptor sizes were similar between the two species, sensory structures were more complex and contain more receptors in the rabbit. These differences in morphological organization may explain an enhanced sensitivity of SAR units to airway pressure in rabbits.

SAR structure and function have been extensively investigated for almost a century. SARs are present in most species examined, including humans (Zhang et al., 2006). The general impression is their structures and electrical activities are similar. By carefully reviewing the literature, it seems that the size of the sensory structure is larger in large animals than in small animals. In other words, sensory structures are simpler in small animals. For example, sensory structures in rats and guinea pigs (Baluk & Gabella, 1991; Matsumoto et al., 2006; Mazzone & McGovern, 2008) look simpler than in rabbits. This is also true when comparing results from the same research group. For example, Yamamoto et al. recognized that sensory structures were more complex in dogs, in which 2–5 different axons may supply a nerve end‐formation that extends up to 400 µm (Yamamoto et al., 1994). Several additional studies indicate that the size of sensory structures are about 100–300 µm in length and 50–100 µm in width in dog (Yamamoto et al., 1995), and about 100–200 µm in length and 50–100 µm in width in the rat (Yamamoto et al., 1999). Sensory unit behavior also differs among species. For example, small animals have a different threshold for SARs (Yu, 2016).

Based on our results, we get the same impression. SAR morphology and behavior, although similar, differ among species. SAR structures appear more complex in rabbits than rats (comparing figures 6 and 8 in Ref (Yu, 2005)). In terms of SAR function, others have reported sensory receptors are more responsive to lung inflation in rabbits than mice. SAR activity roughly increased from 40 to 100 imp/s in rabbits (Lin et al., 2007) and from 50 to 90 imp/s in mice (Zhang et al., 2006) on constant lung inflation pressures of 10 to 30 cmH_2_O, indicating rabbit SAR units are more sensitive to mechanical stimulation. Indeed, our results verify such species differences. Clearly, sensory structures are larger in rabbits than rats. Since their receptor sizes were the same (Figure 2), larger SAR structures are related to more receptors in rabbits (Figures 3, 4, and 5). Greater sensitivity of SARs to lung inflation in rabbits than in rats (Figure 6) is best demonstrated by the steeper regression slope in the rabbits.

In a previous report, we proposed that a different number of sensory receptors in a sensory unit may influence sensory behavior (Liu et al., 2016). Our current data support the hypothesis that greater numbers of SARs in sensory units may contribute to higher activities or sensitivity. The receptor is a basic sensory device that independently generates action potentials. Action potentials are generated from generator potentials, which in turn are determined by the local potential on the sensing surface of the receptor.

Lastly, we address two important questions that the reviewers raised.

Question 1. What is the physiological significance of these species’ differences in SAR units? For example, do they contribute to the expression of the Hering–Breuer reflex (HBR) or are they merely the result of a need for these receptors to innervate a larger area in rabbits than rats?

Answer 1. The Hering–Breuer reflex involves multiple processes involving signal sensing, transduction, and transmission (peripheral and central), including the sensory afferents and synapses, as well as efferent motor neuron and effector function. Expression of the HBR can be potentially affected at any point. Here we only examine the initial segment of the reflex to determine if different species differ in their sensitivity in detecting force. The differences observed between rabbits and rats might explain a high deactivation rate in rabbits (one‐quarter of SAR units deactivated (Guardiola et al., 2014)) compared to rats (personal observation) during constant pressure lung inflation at 30 cm H_2_O, lending support to the hypothesis that there are more SARs in rabbits than rats. More receptors in a unit increase the probability of deactivation. This may also explain why rabbits may have greater strength in the HBR than rats (Widdicombe, 1964). However, such differences do not exclude potential effects arising from other steps in the reflex process. It is also likely that rabbits innervate a larger area than rats; larger animals may have more sensors in a sensory unit. The HBR is a very complex issue. For example, in humans, the sensory signal for the HBR is comparable with animals such as dogs, cats, rabbits, and rats. However, the reflex effect is much weaker probably due to a high central threshold for the reflex. Moreover, our interpretation that large receptor structures may account for higher activity in rabbits is based on correlation. Although logical, cause and effect may not be assumed. Thus, caution needs to be exercised. Nevertheless, the new information is important for understanding the sensory transduction. Currently, we have very limited information at each stage of the reflex, indicating the need for comparative studies.

Question 2. Is it possible some of the structures observed can arise from rapidly adapting receptors or even from sympathetic afferents?

Answer 2. We have identified electronically recorded SAR structures by DiI (1,1′‐dioleyl‐ 3,3,3′,3′‐tetramethylindo carbocyanine) in the rabbit (Wang et al., 2002) and histochemical staining in both rabbit (Yu et al., 2003) and rat (Yu et al., 2004). The SAR structures are plant‐like end‐formations. To date the morphology of RARs has not been characterized, although we believe it is possible current histochemical labeling technique will label them, also. This issue has been addressed in footnote 1 in another paper (Liu et al., 2016). In brief, the vast majority of the structures observed are likely SARs because: (1) SARs significantly outnumber RARs in large airways in a ratio of 4:1 to 10:1(Sant'Ambrogio, 1982); (2) SARs lie in airway smooth muscle of the trachea or larger airway. RARs are thought to distribute around the airway in the epithelium (Sant'Ambrogio et al., 1978). In the current study, we stained tracheal smooth muscle and large airways with the epithelium removed; therefore, it is likely most, if not all, structures are SARs. Regarding the sympathetic afferents, while we cannot exclude the possibility, there are no reports of sympathetic afferents lying in the trachea. Our previous studies identify SARs as plant‐like and leaf‐like end‐formations verified by physiological recording. The chances for vagal and sympathetic structures in the same location and same morphology are slim. With advances in molecular technology, this interesting issue can be further explored through histochemical, genetic, and anatomical identification.

In summary, sensory receptor activation is a very complicated issue. Receptor activation must be influenced by receptor interaction with its surrounding tissues. Unfortunately, these interactions are not very well understood. Thus, all we can say is that the receptor interaction with its environment is important to sensory behavior. On the other hand, receptor morphology inevitably will affect its functional behavior. It should be emphasized that morphological influences should not discount the importance of surrounding tissue interaction. The current studies focused on the morphology issue. We have demonstrated that more receptors in a sensory unit may lead to more sensitivity to a stimulus.

## CONFLICT OF INTERESTS

The authors have no competing interests to declare.

## AUTHOR CONTRIBUTIONS

Study Design: JY. Conducting experiments: PL and JY. Data Acquisition: PL, IZ, and JY. Data Analysis: PL, IZ, and JY. Manuscript writing: PL, IZ, and JY. All authors approved the final version of the manuscript, agree to be accountable for all aspects of the work in ensuring that questions related to the accuracy or integrity of any part of the work are appropriately investigated and resolved. All persons designated as authors qualify for authorship, and all those who qualify for authorship are listed.
